# Distance-Resolving Raman Radar Based on a Time-Correlated CMOS Single-Photon Avalanche Diode Line Sensor

**DOI:** 10.3390/s18103200

**Published:** 2018-09-21

**Authors:** Jere Kekkonen, Jan Nissinen, Juha Kostamovaara, Ilkka Nissinen

**Affiliations:** Circuits and Systems Research Unit, University of Oulu, P.O. Box 4500, 90014 Oulu, Finland; jan.nissinen@oulu.fi (J.N.); juha.kostamovaara@oulu.fi (J.K.); ilkka.nissinen@oulu.fi (I.N.)

**Keywords:** distance-resolving Raman radar, remote Raman spectroscopy, stand-off Raman spectrometer, time interval measurement, time-correlated single photon counting (TCSPC), CMOS single-photon avalanche diode (SPAD)

## Abstract

Remote Raman spectroscopy is widely used to detect minerals, explosives and air pollution, for example. One of its main problems, however, is background radiation that is caused by ambient light and sample fluorescence. We present here, to the best of our knowledge, the first time a distance-resolving Raman radar device that is based on an adjustable, time-correlated complementary metal-oxide-semiconductor (CMOS) single-photon avalanche diode line sensor which can measure the location of the target sample simultaneously with the normal stand-off spectrometer operation and suppress the background radiation dramatically by means of sub-nanosecond time gating. A distance resolution of 3.75 cm could be verified simultaneously during normal spectrometer operation and Raman spectra of titanium dioxide were distinguished by this system at distances of 250 cm and 100 cm with illumination intensities of the background of 250 lux and 7600 lux, respectively. In addition, the major Raman peaks of olive oil, which has a fluorescence-to-Raman signal ratio of 33 and a fluorescence lifetime of 2.5 ns, were distinguished at a distance of 30 cm with a 250 lux background illumination intensity. We believe that this kind of time-correlated CMOS single-photon avalanche diode sensor could pave the way for new compact distance-resolving Raman radars for application where distance information within a range of several metres is needed at the same time as a Raman spectrum.

## 1. Introduction

Raman spectroscopy is a commonly used method for resolving the chemical content of a material. It gives a large amount of information about the molecular structure and chemical environment of the sample and, since pulsed laser technology was introduced, remote Raman spectroscopy devices have also started to be developed e.g., stand-off Raman spectrometers for detecting air pollution and atmospheric backscatter [1,2].

**Figure 1:**
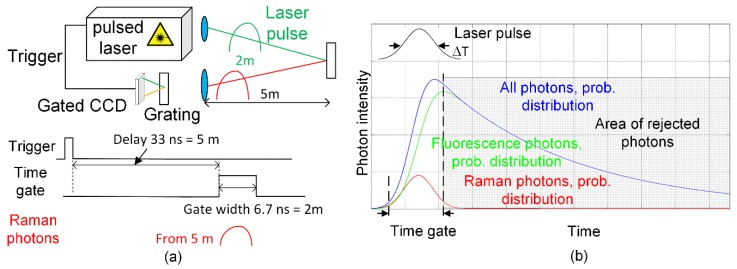
(**a**) Principles of a remote Raman spectrometer based on a pulsed laser and a time-gated charge-coupled device (CCD) and (**b**) fluorescence suppression by time gating.

One of the main problems with stand-off Raman spectrometers when they are used outdoors is the high background radiation that is caused by ambient light. To suppress this background radiation and to allow for the remote detection of the Raman signal, stand-off Raman spectrometers are nowadays based on a pulsed laser and time-gated charge-coupled device (CCD) or intensified charge-coupled device (ICCD) sensors. In such a time-gated system, backscattered Raman photons are collected during the time-of-arrival of an echo pulse from a target sample by enabling detection only during the arrivals of scattered photons from the sample, as illustrated in Figure 1a. This means that the number of background photons can be reduced relative to continuous time measurement. During recent decades, time-gated remote Raman radars have been developed for identifying minerals, explosives and unknown chemical and biological hazardous agents in faraway targets and for planetary exploration [3,4,5,6,7,8]. These remote Raman radars are based on high-power lasers with a pulse width of several nanoseconds and time-gated CCDs or ICCDs with a time gate width varying from several nanoseconds to microseconds, although a time gate width of several nanoseconds may be too long if the background radiation is very high. Besides, a time gate width of several nanoseconds would mean that only metre-level distance resolution would be achievable.

Another major problem in Raman spectroscopy is the fluorescence background of the sample which may mask a weak Raman signal in many applications, e.g., in the identification of explosives and minerals [8,9,10]. The fluorescence background can be suppressed by using a pulsed laser and time-gated CCDs, ICCDs or else a Kerr gate, as illustrated in Figure 1b [11,12]. In such cases, the Raman photons are scattered immediately with the laser pulse, however the fluorescence photons have an exponentially decaying probability density function with a nanosecond or even microsecond lifetime. Thus, the number of fluorescence photons can be dramatically reduced if photons are collected by means of a time-gated detector only during the times of arrival of the Raman photons. These measurement systems are used mainly in laboratories, however, and not in the field, because they are quite expensive, large and complicated. In addition, adequate fluorescence suppression cannot be achieved with a time gate width of several nanoseconds as has been used in previously designed remote Raman spectrometers if the fluorescence lifetime is on a nanosecond scale. Nonetheless, nanosecond-scale fluorescence lifetimes are typical of some applications, as mentioned above, e.g., for identifying explosives, minerals or organic samples [9,10]. Furthermore, some of these applications (e.g., the Mars Rover) call for simple devices, which in practical terms cannot be constructed with time-gated CCDs or ICCDs in view of their power consumption and the achievement of adequate background and fluorescence suppression.

To construct a more practical and compact time-gated fluorescence-suppressed Raman spectrometer, a time-gated single-photon avalanche diode (SPAD) could be used as the detector instead of a CCD or ICCD [13,14]. The probability of the Raman scattering phenomenon is very low, and single photons can be detected digitally by a SPAD because the SPAD acts as a switch during avalanche breakdown. If the photon detection probability in a SPAD-based time-gated Raman measurement is markedly less than one, it will work like any other time-correlated single-photon counting (TCSPC) measurement. In addition, synchronized measurements in which SPADs are biased to the Geiger mode (the mode in which single photons can be detected) only during the laser pulse can be performed “more easily” by using a robust CMOS technology rather than CCDs or ICCDs, whereupon a time gate resolution better than 100 ps can be achieved [13,15]. Moreover, CMOS technology enables one to integrate TCSPC electronics into the same die with a SPAD array, reducing the size and complexity of the whole device [15,16,17,18,19]. The fluorescence suppression effectiveness of a time-gated CMOS SPAD-based Raman microscope has already been proven and it has showed to have potential applications, e.g., in the study of pharmaceutical products [14,20]. Nevertheless, Raman radar devices that are based on a time-correlated CMOS SPAD line sensor that can derive the location of the target sample simultaneously with normal stand-off spectrometer operation, to the best of our knowledge, have not yet been demonstrated and neither has the effectiveness of the fluorescence and background suppression of SPAD-based stand-off Raman spectrometers.

We present here a distance-resolving Raman radar system based on a pulsed laser, a grating and an adjustable time-correlated CMOS SPAD line sensor. The main aim is to show its capability to measure the location of the target sample with centimetre-level resolution while simultaneously obtaining the normal stand-off spectrometer operation. The secondary aim is to show the effectiveness of the background and fluorescence suppression by means of an adjustable time-correlated CMOS SPAD line sensor when measuring a sample without fluorescence and a sample with high fluorescence remotely under different ambient light conditions and at different distances. We believe that this kind of line sensor could pave the way for the development of compact remote Raman spectrometers that could give distance information on targets within a distance range of several meters at the same time as recording the Raman spectrum.

## 2. Raman Radar Set-Up and Test Principles

### 2.1. The Time-Correlated CMOS SPAD Line Sensor-Based Distance-Resolving Raman Radar Device

**Figure 2:**
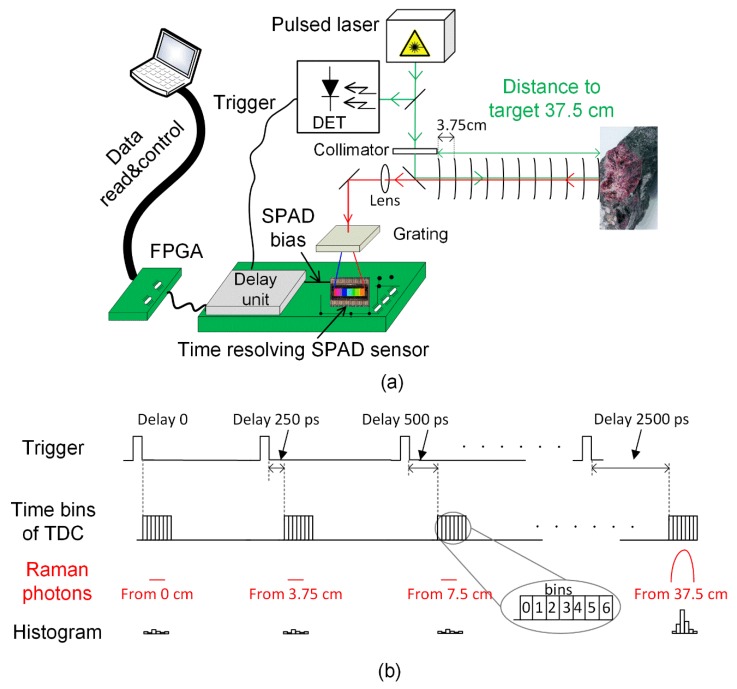
(**a**) Block and (**b**) timing diagrams for the distance-resolving Raman radar based on a time-correlated complementary metal-oxide-semiconductor (CMOS) single-photon avalanche diode (SPAD) sensor.

**Figure 3:**
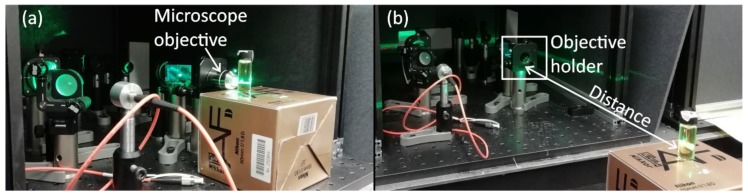
Photographs of the CMOS SPAD-based (**a**) Raman microscope and (**b**) distance-resolving Raman radar.

Block and timing diagrams of the time-correlated Raman radar that was developed in this work are shown in Figure 2a,b, respectively. As shown in Figure 3a, the device was originally designed for measuring samples through the microscope objective that was simply removed to be able to measure samples from longer distances, as shown in Figure 3b. Obviously this is not the most effective way for measuring Raman signals from faraway targets, however the main issue is to show the functionality of the distance-resolving Raman radar based on a time-correlated CMOS SPAD sensor as a proof-of-concept for the purposes of this paper. Photon collection performance can easily be improved by using proper telescope optics for excitation and collection, meaning that much greater distances could be achieved than what are presented in this paper [3,4,5].

The system consists of a 532 nm pulsed laser (Teem Photonics, Meylan, France, model ANG-500P-CHS) as a source of excitation having a pulse width, pulse energy, pulse rate and bandwidth of 160 ps (full width at half maximum = FWHM), 0.6 µJ, 350 kHz and 0.11 nm, respectively, together with optics (beam splitters, mirrors etc.), a diffraction grating, a fast optical detector (DET in Figure 2a), a field-programmable gate array (FPGA) control unit, a digital off-chip delay unit for distance scanning and a 16 × 256 CMOS SPAD-based line sensor with a 256-channel, 3-bit time-to-digital converter (TDC). The pitch of the line sensor is 35.2 µm, resulting in a wavenumber resolution of ~6 cm^−1^ (~0.2 nm). The detailed operation of the 16 × 256 CMOS SPAD-based line sensor with a 256-channel, 3-bit TDC is described in reference [19].

To obtain a Raman spectrum, a sample is excited by a short optical pulse from the laser and simultaneously with the excitation pulse, a synchronization signal (Trigger signal in Figure 2a) is generated optically via a beam splitter by means of a fast optical detector (DET in Figure 2a) in order to bias the SPADs to the Geiger mode in which they are able to detect single photons. To achieve distance scanning, the trigger signal can be delayed in 250 ps steps by means of a digital off-chip delay unit to collect only the photons arriving from the target, thus reducing the effect of the background, as shown in Figure 2a,b, where the distance from the target is 37.5 cm, for example. As the pulsed Raman spectrometer was originally designed to be used for measurements through the microscope objective, this off-chip delay unit was added to the printed circuit board to achieve radar functionality with the previously designed time-correlated SPAD sensor. 

Photon time-of-arrival measurements are started by means of the SPAD bias signal and end with the photons arriving in the bins of the 3-bit TDCs that are located at every spectral point (one channel per spectral point). Time domain histograms can be derived after several thousand laser pulses (histograms in Figure 2b). The bins, numbered from 1 to 6, are stabilized by a reference-locked delay line resulting in a bin size of 100 ps, although bin 0 of the TDCs can be adjusted to be 10 ns by the electrical structure of the time-correlated SPAD sensor. This also allows the collection of the background photons before the target in order to verify the effectiveness of background suppression by comparing the developed sub-nanosecond gating to 10 ns gating which have previously been used with structures based on time-gated CCDs and ICCDs. This is explained in more detail in Section 2.3. An FPGA circuit controls the programming of the detector electronics and reads the measured time domain data from the detector chip. The measured data are post-processed by a Matlab (MathWorks) program to derive the basic Raman spectra and the time domain histograms as a function of the spectral point, for example.

### 2.2. Test Principle for Evaluating the Distance Derivation Capability

Derivation of the distance from the target during stand-off Raman measurement was tested on the basis of the information on the control word of the off-chip delay unit at distances ranging from 15 cm to 40 cm. A titanium dioxide (TiO_2_) sample was placed at distances of 15 cm, 20 cm, 25 cm, 30 cm, 35 cm and 40 cm, which were measured from the front edge of the objective holder (used for microscope objectives) to the front edge of the target, as shown in Figure 3b, using a 5-m measuring tape. At each target distance, the time bins of the TDCs were swept from 15 cm to 41.25 cm by means of an off-chip delay unit with a step size of 3.75 cm (250 ps) to collect photons from different distances, as shown in Figure 2b. In each measurement, 400,000 laser pulses were shot to the target, resulting in acquisition times of approximately 1 s with a pulse rate of 350 kHz.

### 2.3. Test Principle for Evaluating the Effectiveness of Background Supression

**Figure 4:**
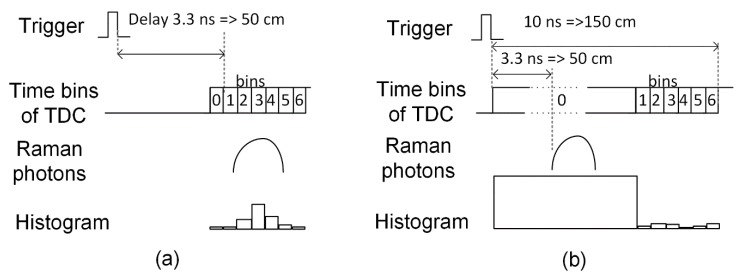
Time gating configurations collecting (**a**) only Raman photons from a target at 50 cm, and (**b**) all photons within a 10 ns time window.

A TiO_2_ sample was used to verify the effectiveness of background suppression in the distance-resolving Raman radar under two sets of background conditions with the laboratory lights on and with an additional halogen lamp used as a background illuminator behind the sample. The background photon count increased by a factor of 30 with the halogen lamp by comparison with the situation in which only the laboratory lights were on. The measured intensities of the illumination at the objective holder were 250 lux (with only the laboratory lights on) and 7600 lux (with the halogen lamp on as well). The distance was increased in 5 cm steps from 15 cm to 500 cm in these tests and the timing was adjusted by means of an off-chip delay unit to collect photons only from the target, as shown in Figure 4a. However, the resolution of the off-chip delay unit (LSB) was 250 ps (3.75 cm), and the nonlinearity of the unit can be a couple of LSBs, especially when longer delays are used, so an additional adjustable coaxial delay unit was provided to achieve 5 cm steps and to compensate for the nonlinearities of the off-chip delay unit. In each measurement, 400,000 laser pulses were shot to the target and efforts were made to distinguish all of the major Raman peaks of TiO_2_ (at 144 cm^−1^, 399 cm^−1^, 516 cm^−1^ and 639 cm^−1^ [21]) in the derived spectra at each distance. In addition, in order to show the effectiveness of the background suppression, the bin zero was adjusted to its maximum value (approximately 10 ns) so that it could collect all of the background within a distance range from 0 to 150 cm, as shown in Figure 4b. The idea of this configuration was to show the difference between the sub-nanosecond time gating that was achieved with the distance-resolving Raman radar that was developed here and the gating of approximately 10 ns commonly used with time-gated CCDs and ICCDs. When distances longer than 150 cm were to be measured, all of the bins (from 0 to 6) were delayed along with the distance for collecting the Raman photons along with the background. The baseline of the dark count noise was removed from the results.

### 2.4. Test Principle for Evaluating the Effectiveness of Fluorescence Suppression

The effectiveness of fluorescence suppression in the distance-resolving Raman radar that was developed here was verified using a sample of olive oil as a target. The Raman spectra of olive oil were measured by adjusting the bins of the TDCs to collect only the Raman photons at distances of 30 cm and 50 cm, and these results were compared with Raman spectra that were measured by the same system but with the zero bin adjusted to its widest value in order to collect the fluorescence tail of the olive oil as well, as shown in Figure 4b. The measured fluorescence lifetime and Raman-to-fluorescence signal ratio of olive oil are approximately 2.5 ns and 33, respectively [22]. During these measurements, 1,000,000 laser pulses were shot at the target.

## 3. Measurement Results

The main aim of this work was to show the distance derivation capability of the distance-resolving Raman radar based on a sub-nanosecond time-correlated 16 × 256 CMOS SPAD sensor. A distance resolution of 3.75 cm was verified by means of measurements within a distance range from 15 cm to 40 cm. In addition, the fluorescence and background suppression effectiveness of the developed Raman radar was shown within a distance range of a few meters. The intensity of the 144 cm^−1^ Raman peak of TiO_2_ is much higher than that of any other Raman peak and, thus, the measured spectra only show the intensities of the Raman peaks with wavenumbers of 399 cm^−1^, 516 cm^−1^ and 639 cm^−1^ to make the spectra more readable.

### 3.1. Results of the Distance Derivation Measurements

**Figure 5:**
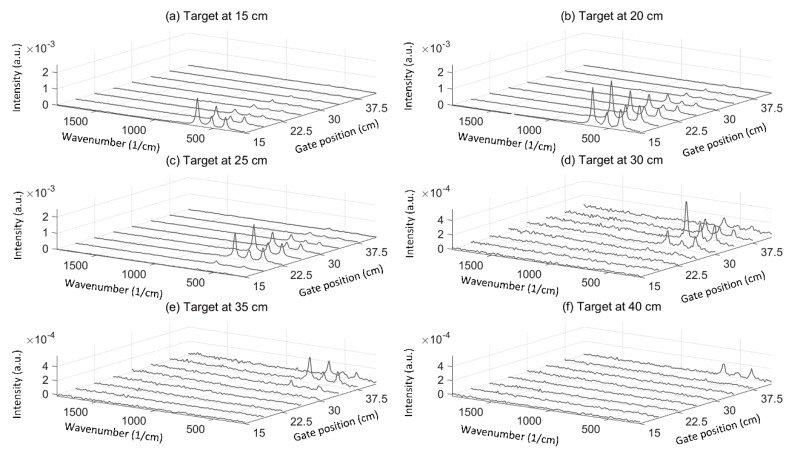
Gate position scanning in 3.75 cm steps with the target at distances of (**a**) 15 cm, (**b**) 20 cm, (**c**) 25 cm, (**d**) 30 cm, (**e**) 35 cm and (**f**) 40 cm.

Figure 5a–f show the Raman spectra of TiO_2_ at distances of 15 cm, 20 cm, 25 cm, 30 cm, 35 cm and 40 cm, respectively, with different gate positions controlled by the off-chip delay unit. The spectra were derived using only the photon count of bin 3 of the TDCs (gate width ~100 ps) to achieve better sampling accuracy than with the hit counts of all of the bins from 1 to 6. The gate position in Figure 5 shows the control word of the delay unit in terms of distances. An overall decrease in the intensity level of the Raman signal can be observed as the distance is increased beyond 20 cm, while the slightly lower intensity of the Raman signal from the target observed at a distance of 15 cm than at 20 cm can be explained by the fact that the maximum intensity of the Raman signal at the 15 cm target distance is located slightly below the 15 cm gate position. In order to show this, the linear range of the off-chip delay unit should have been wider to also measure the previous gate position at 11.25 cm. The range of the off-chip delay unit that was used here to keep the nonlinearities of the delay unit below one LSB was 26.25 cm (only the first three LSB bits were used, which implies eight control words).

**Figure 6:**
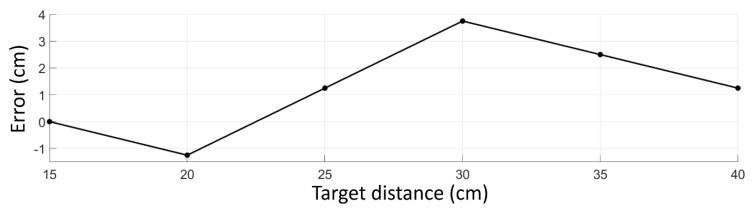
Distance derivation error as a function of the target distance.

As can be seen in Figure 5b–f, the intensity of the Raman signal at target distances greater than 15 cm starts to increase once the gate position is located before the target because it is at this point that bin 3 starts to collect the Raman photons that were generated by the tail of the laser pulse. Nevertheless, the maximum intensity of the Raman signal is observed when the gate position best matches the target distance, as this is where the Raman photons are generated by the peak amplitude of the laser pulse. Note that the impulse response function of the whole system is approximately 230 ps (FWHM), resulting in a total pulse width of approximately 600 ps, corresponding to a distance of 18 cm [19]. The measured target distance was defined to be the gate position where the maximum intensity of the Raman signal was observed. Figure 6 shows the error of the measured target distance as a function of the real target distance. The distance derivation error was measured to be between −1.25 cm and 3.75 cm. The distance derivation error is explained by the nonlinearity and the 3.75 cm resolution of the off-chip delay unit as they cause some error in controlling the gate position.

### 3.2. Results of the Background Suppression Measurements

**Figure 7:**
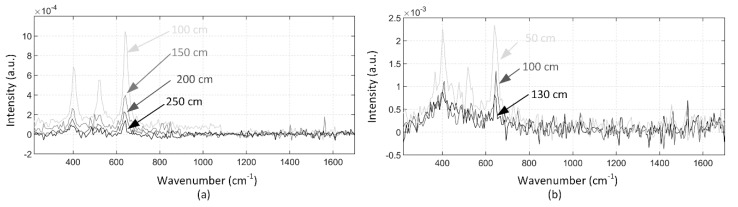
Raman spectra of TiO_2_ measured (**a**) by gating the bins of the time-to-digital converter (TDC) to collect photons from targets at 100 cm, 150 cm, 200 cm and 250 cm, and (**b**) by collecting photons within the 10 ns collection window from targets at 50 cm, 100 cm and 130 cm with the normal laboratory lights on.

**Figure 8:**
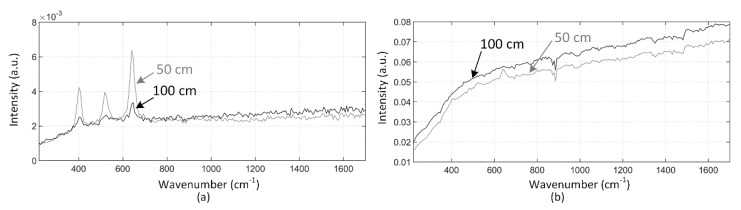
Raman spectra of TiO_2_ measured (**a**) by gating the bins of the TDC to collect photons from targets at 50 cm and 100cm, and (**b**) by collecting photons within the collection window at 10 ns with a halogen lamp shining behind the target.

The Raman spectra of TiO_2_ was measured (a) by gating the bins of the TDC to collect photons from the sample at 100 cm, 150 cm, 200 cm and 250 cm, and (b) by collecting photons within the 10 ns collection window (bins from 0 to 6) with the target at distances of 50 cm, 100 cm and 130 cm, as illustrated in Figure 4a,b, are shown in Figure 7. These measurements were made with the laboratory lights on. The whole Raman spectrum of TiO_2_ is scarcely distinguishable by gating the bins when the target is at 250 cm, however its middle Raman peak starts to vanish below the noise with a 10 ns collection window between distances of 100cm and 130 cm. In addition, the same measurement was performed with a higher background radiation which was enabled by using a halogen lamp behind the TiO_2_ sample at 50 cm and 100 cm. The photon detection probability was then increased by a factor of approximately 30, relative to the previous measurement, as seen from the Raman spectra that is shown in Figure 8a (gated at the target) and Figure 8b (10 ns collection window). Now all of the Raman peaks of TiO_2_ can be distinguished from the spectra measured by gating the bins at the target, as shown in Figure 8a, however only the highest peak of the spectrum can be distinguished by using the 10 ns collection window when the target is at 50 cm, as shown in Figure 8b. When the target is moved to 100 cm, the two strongest Raman peaks can be clearly distinguished from the spectra that was measured by gating the bins at the target, however none of the peaks can be distinguished when using the 10 ns collection window. The decrease in the background level between distances of 50 cm and 100 cm can be explained by the position of the target and the target holder, which were shadowing the halogen lamp radiation more when the target was at 50 cm. These measurement results clearly show the advantage of the sub-nanosecond collection window that was used here over the 10 ns collection window that is used in many stand-off Raman devices based on time-gated CCDs and ICCDs with high background radiation.

**Figure 9:**
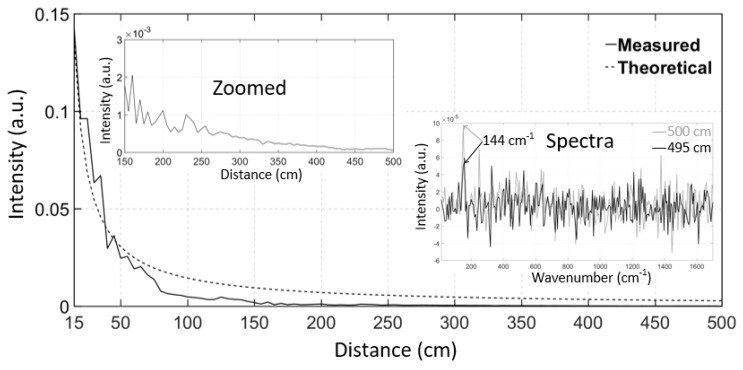
Intensity of the 144 cm^−1^ Raman peak of TiO_2_ as a function of distance, from 15 cm to 500 cm (solid line), and a theoretical curve based on the radar equation (dashed line) and Raman spectra at distances of 495 cm and 500 cm.

The whole Raman spectrum of TiO_2_ could not be distinguished at distances longer than 250 cm, however the occurrence of the strongest Raman peak at a wavenumber of 144 cm^−1^ could be distinguished even at a distance of 500 cm (with the laboratory lights on). The intensity of this peak (solid line) and a theoretical curve that is based on the radar equation (dashed line) as a function of distance are shown in Figure 9. This figure also shows the zoomed data from 150 cm to 500 cm in order to demonstrate the fluctuation in the measured intensity at longer distances, which is not visible in the full-sized figure, and the Raman spectra at distances of 495 cm and 500 cm. The intensity follows the radar equation quite well as it is inversely proportional to the square of the distance. The measured intensities had slightly lower values than the radar equation when longer distances were measured, which can be explained by the fact that the spot of the laser started to exceed the size of the target.

### 3.3. Results of the Fluorescence Suppression Measurements

**Figure 10:**
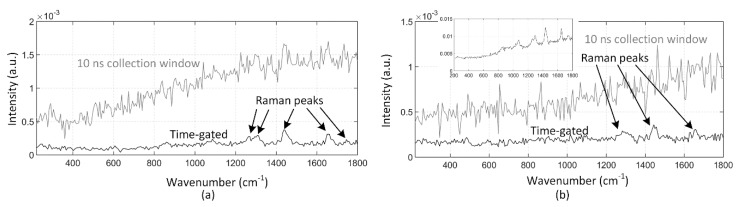
Raman spectra of olive oil measured by gating the bins of the TDC to collect photons from the target and collecting photons within a 10 ns collection window at distances of (**a**) 30 cm and (**b**) 50 cm with the normal laboratory lights on.

The effectiveness of fluorescence suppression in the time-correlated CMOS SPAD sensor-based distance-resolving Raman radar was verified by measuring a sample with high fluorescence with the laboratory lights on. The Raman spectra of olive oil that were measured by gating the bins of the TDC to collect the photons only from the target and by collecting photons within the 10 ns collection window when the target was at (a) 30 cm and (b) 50 cm are show in Figure 10. As can be seen, the Raman peaks of olive oil cannot be clearly distinguished using the 10 ns collection window at either distance. A small panel on the top of Figure 10b shows the Raman spectrum of olive oil measured through the microscope objective as a reference [19]. All of the major Raman peaks that are used for the authentication and analysis of fat content in food oils can be distinguished in the spectra measured by gating the bins at the distance of 30 cm, and the highest peaks can be distinguished even at 50 cm [23]. These results also clearly show the advantage of the sub-nanosecond collection window that was used here over the conventional 10 ns collection window when a sample with high fluorescence is being measured.

## 4. Discussion and Conclusions

This paper presents a distance-resolving Raman radar based on a pulsed laser, a 16 × 256 CMOS SPAD line sensor with a 256-channel TDC and a digital off-chip delay unit. The technology concerned enables distance scanning with 3.75 cm resolution in order to derive the distance from the Raman sample simultaneously with the Raman spectrum. The CMOS technology makes it possible to integrate optical detectors and all the other electronics into the same die and, thus, sub-nanosecond gating can be achieved more conveniently than with traditional time-gated CCDs or ICCDs, in which gating of several nanoseconds is normally used. This allows us to further reduce the background noise and the fluorescence of the sample that has a nanosecond-scale lifetime. We believe that this technology could pave the way for a new kind of distance-resolving Raman radar device for applications in mining and explosive detection, where a compact stand-off device with a distance range of several metres is needed.

Since the optics and SPAD sensor circuit of the Raman radar that was used here were originally optimized for measurements through the microscope objective with the same excitation and collection axis, the objective of the microscope were simply removed and the measurements were performed at what was optically a relatively poor performance level. In addition, the off-chip delay unit was needed here because cm-level gate position sweeping of this kind had not been needed in the previously designed Raman microscope. Our main aim here was to show the distance derivation capability and the effectiveness of the fluorescence and background suppression, Which was achieved with the CMOS technology-based SPAD line sensor as a “proof-of-concept” in a stand-off Raman radar. Photon collection efficiency could be improved by proper designing of the optics, and this will be done in the future. Future work will also concentrate on SPAD line sensor development in order to increase the level of integration of the line sensor to enable cm-level gate position scanning to be performed by means of an on-chip circuit. A stabilized delay unit with a smaller LSB for distance scanning will be integrated into the same die to achieve more stable, better resolution and smaller nonlinearities than with a commercial off-chip delay unit.
